# Bidirectional pathogenesis between non-tuberculous mycobacteria and bronchiectasis: clinical insights, diagnostic challenges and future directions—Perspectives from South Asia

**DOI:** 10.3389/ftubr.2025.1735568

**Published:** 2026-01-12

**Authors:** Amresh Kumar Singh, Sneha Singh, Nandini Singh, Priyanka Gaur, Ashwini Kumar Mishra, Raj Kishore Singh, Sushil Kumar

**Affiliations:** 1 Department of Microbiology, Baba Raghav Das Medical College, Gorakhpur, India; 2 Department of Zoology and Environmental Science, Deen Dayal Upadhyaya Gorakhpur University, Gorakhpur, India; 3 Department of Zoology, Deen Dayal Upadhyaya Gorakhpur University, Gorakhpur, India; 4 Department of Tuberculosis & Chest, Baba Raghav Das Medical College, Gorakhpur, India; 5 Department of Medicine, Baba Raghav Das Medical College, Gorakhpur, India

**Keywords:** bronchiectasis, diagnosis dilemma, non-tuberculous mycobacteria, post-tuberculosis, South Asia

## Abstract

Non-tuberculous mycobacteria (NTM) are environmental opportunistic pathogens causing chronic pulmonary disease. NTM pulmonary disease (NTM-PD) and bronchiectasis exhibit a bidirectional pathogenic relationship that is particularly under recognized in South Asian regions with high tuberculosis (TB) burden. This review article covers most of the current evidence on the epidemiology, clinical spectrum, pathogenesis, therapeutic advances of NTM-associated bronchiectasis. A well-structured literature search was conducted across PubMed, Web of Science, Scopus, and Google Scholar for articles published within from 2008 to 2025. These studies were specifically focused on NTM -associated bronchiectasis for host pathogen interactions, diagnostic strategies, and treatment outcomes. The prevalence of NTM related bronchiectasis especially with species like *Mycobacterium avium* complex and *Mycobacterium abscessus* which were more frequent pathogens all over the world. Its diagnostic dilemma with TB remains widespread due to limited lab capacity and lack of exact species identification, which leads to late or wrong diagnosis. Integration of molecular diagnostic tools, inclusion of NTM within national TB programs, and establishment of regional reference laboratories are essential to improve early detection, targeted treatment, and disease surveillance for bronchiectasis.

## 1 Introduction

Non-tuberculous mycobacteria (NTM) are ubiquitous environmental organisms found in water, soil, and dust. More than 200 species have been identified up to till date although only a subset cause human disease (1). Over the last two decades, the global prevalence of pulmonary NTM disease has steadily increased, largely due to enhanced awareness, improved diagnostic techniques, an aging population, and the rising incidence of chronic lung diseases such as bronchiectasis and chronic obstructive pulmonary diseases (COPD) (2). Epidemiological studies show that the prevalence of NTM related bronchiectasis is about 10% in adults globally, in which *Mycobacterium avium* complex (MAC) is the most frequently isolated pathogen (3). Other clinically relevant species include *M. abscessus, M. kansasii*, and *M. simiae* (4, 5). NTM was initially thought to be an environmental organism but later gained importance as a potential opportunistic pathogen associated with both pulmonary and extrapulmonary infection (6, 7).

Pulmonary NTM infections and bronchiectasis are intricately linked and they exist as both cause and consequence of each other (8). NTM species, such as MAC, *M. abscessus*, and *M. kansasii*, cause chronic lung infections presenting with persistent cough, fatigue, and systemic symptoms (9). These infections flourish in structurally damaged lungs, where weakened mucociliary clearance and chronic inflammation helps to facilitate colonization of bacteria and disease progression. NTM infections significantly worsen respiratory outcomes in cystic fibrosis (CF) and other chronic pulmonary diseases and can be relative contraindications for lung transplantation (10). Increase in NTM prevalence shown by epidemiological trends have highlighted the growing clinical significance of these opportunistic pathogens (11).

Bronchiectasis act as both a risk factor for and a consequence of NTM infection, showing a bidirectional relationship (4, 12). Although, the bronchiectasis is well recognized, the clinical spectrum of disease remains heterogeneous and distinguishing colonization from true infection is still challenging (1). Post-tuberculosis lung diseases (PTLD) have emerged as a structural risk factor for NTM- pulmonary disease (NTM-PD) especially in TB endemic area like Asia. PTLD commonly results in fibrosis, cavitation, and traction bronchiectasis, all of which create a chronic niche that favors NTM colonization (13). Recent population-level assessments further demonstrate that a substantial proportion of TB survivors continue to experience long-term respiratory impairment, highlighting a growing burden of post-TB sequelae in Asia (14). Structural damage following healed TB has also been identified as one of the strongest predisposing factors for NTM infection across multiple cohorts, reinforcing its mechanistic relevance in disease susceptibility (15).

This review basically aims to synthesize current evidence on the epidemiology and pathogenesis of NTM-associated bronchiectasis, also to highlight diagnostic challenges specific to high TB- burden Asian settings and identify gaps in surveillance, diagnosis, and management requiring urgent attention. Our review focused on misdiagnosis and delayed management for NTM infections which can lead in rising global prevalence of NTM-associated bronchiectasis and data from high-burden, TB endemic regions has confirmed it. Our article aims to bridge this knowledge gap and highlight region-specific considerations. This article also emphasizes the urgent need to fulfill the diagnostic gap by enhancing diagnostic capacity, species-level surveillance, and targeted therapeutic strategies to improve patient outcomes and guide clinicians and researchers toward more precise, effective efforts to manage NTM-associated bronchiectasis.

## 2 Data sources and search strategy

This review was conducted to summarize and interpret the current evidence on the bidirectional relationship of NTM pulmonary disease (NTM-PD) and bronchiectasis, with a particular focus on Asian countries. Literature search was done by using major scientific databases, including PubMed, Scopus, Web of Science and Google Scholar covering articles published from 2008 to 2025. The search strategy combined Medical Subject Headings (MeSH) and keywords: (“nontuberculus mycobacteria” OR “NTM” OR “atypical mycobacteria”) AND (“bronchiectasis”) AND (“pathogenesis” OR “treatment” OR “epidemiology”). For regional focus, we added: (“India” OR “South Asia” OR “Asia-Pacific”). These were included to capture relevant studies which show regional prevalence and diagnostic challenges.

These included studies were selected on the basis of their relevance to NTM-associated bronchiectasis and data describing pathogenesis, epidemiology, diagnostic methods, treatment outcomes, and disease prognosis were extracted. References from the key were further screened to identify additional literature not retrieved during the initial search. Articles focused exclusively on *Mycobacterium tuberculosis* complex (MTBC) or extrapulmonary NTM infections were excluded.

## 3 Clinical spectrum and disease manifestations

NTM and bronchiectasis are closely related as they can lead to each other and result in bronchiectatic illness. MAC and *M. abscessus* are the most isolated in bronchiectasis-associated NTM-PD, even though their epidemiology is confounded by biases in detection and reporting. NTM-PD infections are clinically presented as highly variable and often non-specific. Its common symptoms include chronic cough, sputum production, fatigue, losing weight, hemoptysis, dyspnea, and night sweats (16). NTM-PD commonly showed nodular/bronchiectatic and fibro-cavitary (FC) forms being the primary manifestations (17). NTM disease involves tree-in-bud opacities, pulmonary cavitary lesions, nodules, and mucus plugging, with frequent involvement of upper lobes and multiple lung lobes (18). Clinical presentation may mimic other chronic lung conditions, which can delay diagnosis (19). For example, one of the disease as The Lady Windermere syndrome caused by MAC, is often seen in elderly women without underlying structural lung disease (20).

*M. abscessus* forms biofilm which further complicates disease progression by enhancing antibiotic tolerance and immune evasion (21). It forms chronic inflammation and repeated infections result in progressive bronchiectasis and irreversible airway damage (17). There is subtle appearance of symptoms in early disease but further these symptoms proliferate with chronic infection which causes significant morbidity. Two major patterns are recognized radiologically. First one is nodular/bronchiectatic form which is the most frequent and typically affecting thin, older, non-smoking women. It is also often localized to the middle lobe or lingula, and associated with multifocal bronchiectasis and small nodules (1, 4). This form progresses slowly but can lead to cumulative lung damage. The second form is FC disease, which involves upper lobes, resembles pulmonary TB, and is associated with a higher bacterial burden and faster progression, often occurring in older men with COPD or a smoking history (5).

Other less common presentations of bronchiectasis include solitary pulmonary nodules, extensive or disseminated disease, and hypersensitivity-like pneumonitis (4). Disseminated disease is rare but occurs in immunocompromised patients, while skin, bone, and lymph node involvement have been reported (22). Radiological severity scores and features such as tree-in-bud pattern, multilobar involvement, and bilateral disease are notable predictors of NTM positivity in bronchiectasis cohorts (23). Co-infections with pathogens like *Pseudomonas aeruginosa, Staphylococcus aureus*, and *Acinetobacter baumannii* are frequent and further complicate disease course (4). Overall, the spectrum of NTM-PD ranges from indolent nodular disease to aggressive cavitary and disseminated forms, and clinical manifestations must always be interpreted alongside radiological and microbiological findings to confirm true disease.

## 4 Regional perspective

### 4.1 Global epidemiology

The global burden of NTM-PD has shown a significant upward trend over the past decades. In the United States, the annual incidence of NTM lung disease increased from 3.13 per 100,000 person-years in 2008 to 4.73 per 100,000 in 2015, and prevalence rose from 6.78 to 11.70 per 100,000 over the same period (10). A comprehensive review of NTM-PD epidemiology across more than 18 countries found that most studies reported rising rates of NTM isolation approximately 82% and NTM disease approximately 67%, with the MAC being the predominant species (11). In Hawaii, prevalence increased from 20 to 44 per 100,000 between 2005 and 2013, while Florida showed growth from 14.3 to 22.6 per 100,000 between 2012 and 2018 (11). Regional variations are striking-a Delphi survey reported prevalence rates of 6.1–6.6 per 100,000 in European countries, but ~25 per 100,000 in Japan, a fourfold difference (11). Globally, a meta-analysis in CF populations estimated a pooled NTM infection prevalence of 7.9% (95% CI: 5.1–12.0%), with an increasing trend between 2010 and 2019 (10). The prevalence of NTM-PD in Africa ranges from 0.2% to 28% across different studies, with the highest reports from South Africa, Ethiopia, and Nigeria (24). The most isolated species in these types of cases are MAC, *M. fortuitum*, and *M. abscessus*, which together account for most cases. A study in Denmark on HIV-positive individuals have shown that MAC alone represents up to 50% of NTM infections in them (25).

These data highlight a steadily rising global disease burden with significant geographic heterogeneity and species diversity. In a comprehensive analysis of global registry data, the prevalence of NTM-PD in bronchiectasis was estimated at 10.0% (95% CI: 6.0–14.0%), with MAC accounting for 65–90% of isolates. Environmental factors such as humidity, water sources, and aerosol exposure significantly influenced disease distribution (Table 1).

**Table 1:** Epidemiology and major findings of NTM and bronchiectasis interactions (global epidemiology).

**Study**	**Region/country**	**Study population**	**NTM prevalence/isolation rate**	**Predominant NTM species**	**Bronchiectasis association**	**Key findings**
Prevots, 2023	Global (Asia, Africa, Americas)	Review of population-based NTM data	15% among TB suspects in Asia; up to 20% in MDR-TB	MAC predominant; regional variation	High risk in patients with structural lung disease, esp. bronchiectasis	Environmental and host factors drive geographic variability; humid climates increase risk (11)
Martinez-Garcia, 2024	Israel/Global Review	Bronchiectasis patients with NTM	18% NTM positivity among bronchiectasis patients	MAC, *M. simiae, M. kansasii, M. abscessus*	NTM common in severe, multilobar bronchiectasis with low BMI	Species-specific prognosis; RGM linked to poor outcomes; MAC to chronic progression (26)
Prados Sánchez, 2016	Spain (Global overview with Asian data)	Comprehensive review of bronchiectasis-NTM cases	30% prevalence among bronchiectasis cohorts worldwide	*M. avium* complex, *M. abscessus* complex, *M. gordonae*	Five clinical types of NTM-bronchiectasis; radiologic and microbiologic overlap	Clinical phenotypes defined: fibrocavitary, nodular, solitary, hypersensitivity, disseminated (4)
Daley, 2020	Global (RCT populations, USA, Europe, Asia)	Refractory NTM-PD patients in phase III trials	20% refractory NTM-PD rate despite guideline therapy	*M. avium* complex predominant; RGM in refractory cases	Refractory cases often have underlying bronchiectasis	Inhaled amikacin effective in refractory bronchiectasis-NTM patients (35)
Gardini, 2021	Global review (Environmental epidemiology)	Environmental samples, household & hospital settings	Environmental prevalence up to 45% in water systems	*M. avium* complex, *M. gordonae, M. fortuitum* common in water systems	Environmental exposure higher in bronchiectasis households	NTM distribution strongly linked to water sources and regional humidity (34)
Cowman, 2019	Europe/Global	Multicenter observational studies on NTM-PD patients with chronic lung disease	NTM detected in 10–25% of bronchiectasis patients	MAC, *M. abscessus, M. xenopi*	Bronchiectasis present in >50% of NTM-PD cases	Bronchiectasis severity correlated with higher exacerbation frequency, persistent NTM infection, and poorer outcomes (32)
Philley, 2016	USA (Single-center cohort)	168 NTM-PD patients with and without bronchiectasis	NTM-PD prevalence 18% among chronic lung disease patients	*M. abscessus*, MAC*, M. kansasii*	Bronchiectasis increased risk of chronic colonization and biofilm formation	Biofilm-mediated persistence contributed to treatment failure and relapse; impaired clearance major risk factor (52)
Dhasmana, 2024	UK/Europe	Review of chronic NTM-PD with airway disease	14–22% prevalence in bronchiectasis cohorts	MAC*, M. kansasii, M. gordonae*	NTM infection often coincided with worsening bronchiectasis and lung function decline	Highlighted importance of immunological impairment and structural changes in sustaining NTM colonization (55)
Chalmers, 2018	UK/Europe	Prospective bronchiectasis cohort (n=143)	NTM identified in 15.7% of patients	MAC, *M. abscessus*	Bronchiectasis severity correlated with NTM burden	Chronic neutrophilic inflammation and repeated exacerbations contributed to progressive airway damage and increased NTM persistence (54)
Larsson, 2017	Sweden/Europe	Global review of NTM infection mechanisms	Reported NTM isolation in up to 30% of bronchiectasis cases globally	MAC*, M. abscessus, M. chelonae*	Structural lung damage and impaired mucociliary clearance linked to NTM colonization	Emphasized environmental exposure, defective immune response, and structural remodeling as major pathogenic drivers (51)

### 4.2 Asian epidemiology

The interplay between bronchiectasis and NTM disease in Asian nations with the presence of humid environment, and limited diagnostic infrastructure, presents a distinctive epidemiological and clinical pattern shaped by the region's high TB burden. We have summarized the data of different Asian studies focussed on their prevalence and key findings (Table 2). Unlike Western populations, where NTM-associated bronchiectasis often arises in elderly, thin, non-smoking women with idiopathic or MAC-related disease, the South Asian phenotype is predominantly post-TB bronchiectasis with secondary NTM infection (12, 26).

**Table 2:** Epidemiology and major findings of NTM and bronchiectasis interactions (Asian epidemiology).

**Study**	**Region/country**	**Study population**	**NTM prevalence/isolation rate**	**Predominant NTM species**	**Bronchiectasis association**	**Key findings**
Suresh, 2021	Kerala, India	7,073 TB suspects (pulmonary + extrapulmonary)	1.4% overall (14.8% of mycobacterial isolates)	*M. abscessus* (33.3%), *M. fortuitum* (24.6%)	Post-TB bronchiectasis major risk factor (16.2%)	High species diversity; diabetes, malignancy, prior TB common comorbidities (28)
Rout, 2025	Odisha, India	828 suspected TB/bronchiectasis patients	8.1% of TB-suspect samples positive for NTM	*M. intracellulare* (32.8%), *M. abscessus* (22.3%), *M. fortuitum* (11.9%)	Strong link with post-TB and chronic bronchiectasis	Highest prevalence in humid coastal regions; male predominance (65.7%) (30)
Simons, 2011	Asia (India, China, Japan, Korea, SE Asia)	1,744 Asian patients with pulmonary NTM	31% of isolates clinically relevant (NTM-PD)	MAC (68%), RGM (14%), *M. abscessus, M. fortuitum, M. chelonae*	44% of cases had bronchiectasis; 37% had prior TB	Asian pattern: male predominance, post-TB background, RGM dominance (27)
Fujita, 2025	Japan/Asia-Pacific	Narrative review of regional registries and cohort data	10–20% of bronchiectasis patients positive for NTM	MAC, M. abscessus complex	Bidirectional association confirmed; NTM also induced bronchiectasis progression	Structural airway damage, mucus retention, and immune dysregulation enhance susceptibility to NTM colonization (9)
Choi, 2024	Korea, Japan, Asia-Pacific	Bronchiectasis registries and national cohort data	20% among bronchiectasis patients in Asia	MAC and *M. abscessus* most frequent	Post-infectious bronchiectasis dominant in Asia (vs idiopathic in West)	Cavitary, tree-in-bud, multilobar disease patterns predict NTM positivity (12)
Wang, 2024	Taiwan (Clinical predictors study)	Bronchiectasis cohort with NTM infection (n=429)	16.4% of bronchiectasis cohort NTM-positive	*M. intracellulare, M. abscessus, M. kansasii*	Tree-in-bud, cavitary lesions, multilobar disease predict NTM	Hemoptysis, post-infectious etiology, high radiologic scores linked to NTM (23)
Pathak, 2022	India (Review focus, global comparison)	Clinical and radiological cases of NTM lung disease	15% of chronic lung disease patients positive for NTM	*M. avium* complex, *M. abscessus, M. kansasii*	Radiological overlap with bronchiectasis; nodular and cavitary forms common	Detailed diagnosis, imaging differentiation from TB emphasized (1)

Asian meta-analyses further corroborate this relationship by multiple studies. A literature-based study of 1,744 pulmonary NTM cases across eastern Asia had shown 37% had a prior history of TB (27). Structural lung abnormalities, including bronchiectasis and nodular changes, were highlighted as key features in these patients, underscoring the importance of bronchiectasis in the pathogenesis of NTM lung disease. In this cohort study, *MAC* caused 68% of cases, but rapidly growing mycobacteria (notably *M. abscessus* and *M. fortuitum*) accounted for 14%, especially in India, Taiwan, and southern China. The recent reviews also emphasized that the Asian bronchiectasis-NTM phenotype is dominated by post-infectious (especially post-TB) etiologies (12, 26). Across Asia, NTM prevalence among bronchiectasis cohorts ranges from 9 to 20%, compared to < 5% in Western registries. In Korea and Japan, Choi, 2024 estimated that up to one in five bronchiectasis patients develop NTM-PD, with MAC and *M. abscessus* as leading pathogens (12). The presence of cavitary lesions, tree-in-bud pattern, and multilobar involvement strongly correlated with NTM positivity-findings echoed in Indian cohorts.

Recent evidence indicates a rising prevalence of NTM-PD among bronchiectasis cohorts in India. In a multi-year hospital-based study from Kerala, reported that among 7,073 TB-suspect samples, 1.4% were NTM-positive, constituting 14.8% of all mycobacterial isolates (28). Of these, 40% were pulmonary, and nearly 16% of patients had prior TB, confirming that post-TB lung damage is a major precursor of NTM-associated bronchiectasis. *M. abscessus* (33.3%) and *M. fortuitum* (24.6%) dominated, with 65.7% of isolates being rapidly growing mycobacteria (RGM), a trend consistent with earlier South Indian series where RGM predominated over MAC (28). In eastern India, researchers analyzed over 800 clinical isolates from TB-suspect patients and reported 8.1% culture positivity for NTM, again primarily in individuals with post-TB bronchiectasis and chronic sputum production (Table 2) (29). The *M. intracellulare, M. abscessus*, and *M. fortuitum* were leading pathogens, while co-infection with *Pseudomonas aeruginosa* was common-an important marker of advanced bronchiectatic disease (30). The highest positivity was recorded in humid coastal districts, implicating environmental exposure from household and soil sources.

From an ecological and clinical perspective, India's high post-TB bronchiectasis burden provides a fertile environment for NTM colonization. Warm, humid climates, coupled with biofilm formation in domestic water systems, favor persistence of RGM species might be a reason of high burden (11). Structural lung changes from healed TB (fibrosis, traction bronchiectasis, and destroyed lobes) impair mucociliary clearance, allowing opportunistic NTM to establish chronic infection. This explains why Indian NTM-bronchiectasis patients often present at a younger age (40–60 years) than their Western counterparts with true underestimated epidemiological burden (28). In India, diagnostic confusion with TB remains pervasive due to reliance on smear microscopy and CBNAAT, which cannot differentiate *M. tuberculosis* from NTM. Consequently, many bronchiectasis patients with NTM-PD receive prolonged or repeated anti-tubercular therapy, delaying appropriate management. Most laboratories lack capacity for species-level identification and drug susceptibility testing, resulting in incomplete surveillance. National estimates suggest that 1–10% of “treatment-failure TB” or “recurrent bronchiectasis” cases are undiagnosed NTM disease.

## 5 Risk factors

Multiple risk factors predispose individuals to NTM related diseases. At the host level, advanced age, female sex, low body mass index, anemia, and hypoalbuminemia have been consistently associated with NTM positivity (5, 11). Immune dysregulation, including HLA defects and impaired macrophage function, increases susceptibility, while systemic immunosuppression due to diabetes, malignancy, or transplantation further enhances risk (4). A Korean nationwide cohort confirmed that bronchiectasis itself is a strong independent risk factor, conferring a nearly 19-fold increased risk of NTM-PD compared with the general population (12).

Patients with NTM must exhibit new or worsening pulmonary symptoms, radiographic evidence (nodules, cavitary lesions, or bronchiectasis), and microbiologic confirmation (positive cultures from sputum or bronchoalveolar lavage, or granulomatous histology) (19). Risk factors include chronic lung diseases such as COPD, CF, interstitial lung disease, and non-CF bronchiectasis. Older age, female sex, low body mass index, gastroesophageal reflux disease, and greater lobar involvement are significantly associated with NTM infection (17). Ciliary dysfunction, whether primary (genetic) or secondary (post-infectious, smoking-related), impairs mucociliary clearance and increases susceptibility to NTM (20).

Prior TB also remains one of the strongest predisposing factors for NTM-PD in TB endemic regions. This is largely due to residual structural abnormalities such as fibrosis, cavitation, and airway remodeling. In such condition, the fibro cavitary pattern is commonly observed and reflects a form of post-TB lung damage that compromises mucociliary clearance and creates an environment conducive to microbial persistence (31, 32). This pattern may occur alongside or evolve into bronchiectasis through chronic inflammation, airway instability, and progressive epithelial injury. Studies from Asian cohorts further support this association, demonstrating that fibro cavity post TB disease significantly increases vulnerability to NTM infection compared to individuals with structurally normal lungs (9, 12). Thus, in high TB-burden regions, fibro cavitary sequelae represent a key risk factor for NTM-PD and help explain the characteristic radiological phenotypes observed in South Asian patients.

At the pulmonary level, prior history of TB, COPD and structural bronchiectasis are recognized as major risk factors for NTM (29, 33). Radiological features such as tree-in-bud opacities, multilobar disease, and cavitary lesions also correlate with higher risk of NTM (5, 23). Long-term macrolide prophylaxis in bronchiectasis patients, while effective in reducing exacerbations, has been linked to higher incidence of NTM disease, likely through selective antimicrobial pressure (12). Environmental exposure is central to pathogenesis, especially household water systems, biofilms in showers, indoor swimming pools, humidifiers, and soil are important reservoirs of NTM (34). Aerosolized exposure from these niches has been epidemiologically associated with pulmonary disease, and even genetic matches have been demonstrated between patient isolates and household strains. This highlights the interplay between environment, host vulnerability, and structural lung damage in the development of disease.

## 6 Diagnosis of NTM

**Figure 1:**
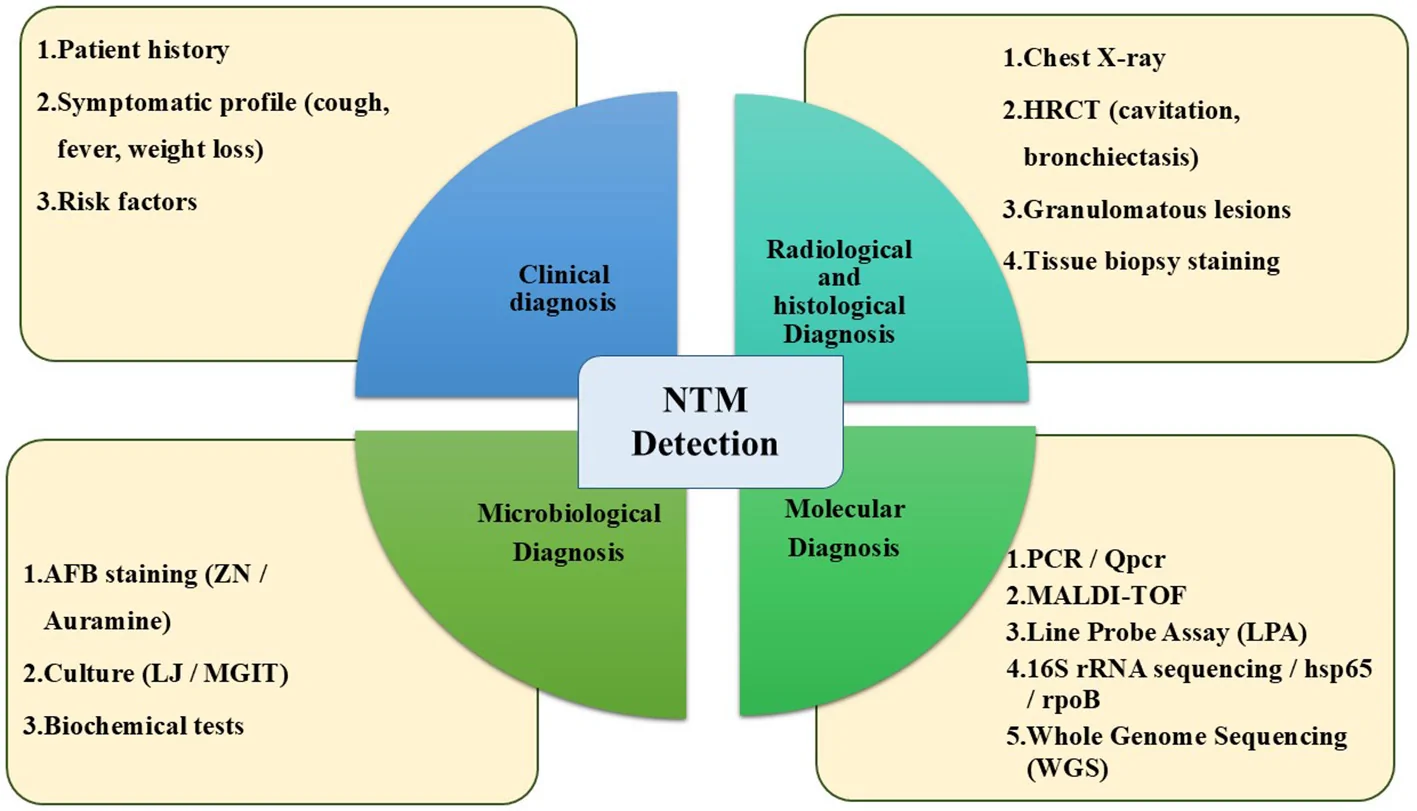
Different diagnostic approaches for NTM Detection across four diagnostic categories. NTM, Non-tuberculous Mycobacteria; HRCT, High-Resolution Computed Tomography; AFB, Acid-Fast Bacilli; ZN, Ziehl-Neelsen; LJ, Löwenstein-Jensen; MGIT, Mycobacteria Growth Indicator Tube; PCR, Polymerase Chain Reaction; qPCR, Quantitative Polymerase Chain Reaction; hsp65, Heat shock protein 65 gene for species identification of mycobacteria; *rpo*B, RNA polymerase $\beta$ subunit gene for species identification and detection of drug resistance (35, 36, 40).

The diagnosis of NTM pulmonary disease remains complex because NTM can exist as mere colonizers in damaged lungs or as true pathogens causing progressive disease. According to international guidelines, confirmation requires a combination of clinical symptoms, radiological abnormalities, and microbiological evidence, with at least two positive sputum cultures or one positive bronchoscopy sample as mentioned in Figure 1. Diagnostic challenges persist due to overlapping symptoms with other lung diseases and heterogeneity in clinical markers (17). Diagnosis requires a combination of clinical, radiological, and microbiological criteria, including positive cultures from sputum or bronchoalveolar lavage and imaging findings such as nodular bronchiectatic or FC patterns (8). It symptoms overlap with other lung diseases, repeated cultures and molecular testing are often essential for confirmation (35). Molecular assays and sequencing are increasingly used for rapid species-level identification, although culture remains the gold standard for NTM (36). Distinguishing colonization from active infection is crucial, as unnecessary treatment exposes patients to toxic multidrug regimens, while delayed therapy risks irreversible lung damage (4).

**Figure 2:**
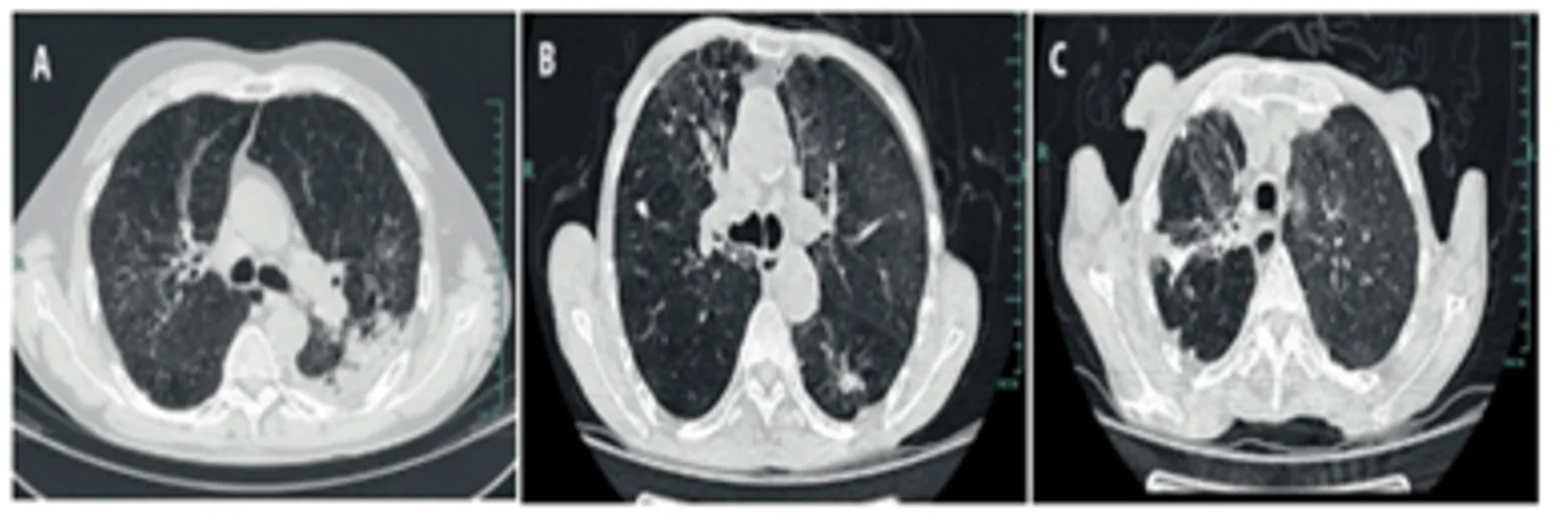
Chest CT imaging showed. **(A)** Chest CT image in NTM group. The two lungs showed multiple spotted patchy nodules and shadows, especially in the lower lobes, which were scattered in smooth thin-walled cavities with various shapes and sizes. **(B)** Chest CT image in active TB group. Multiple micronodules in the middle and lower lobe of the right lung were clustered together, accompanied by thickened interlobular septum. There were micro-nodules in the center of the lobules and tree buds in the upper lobes of both lungs. **(C)** Chest CT image in MDR-TB group. There were nodular and patchy high-density shadows in the upper lobes of both lungs. The upper right lobe exhibited contractile changes, thickening of the pleura on both sides, and a small amount of pleural effusion.

**Figure 3:**
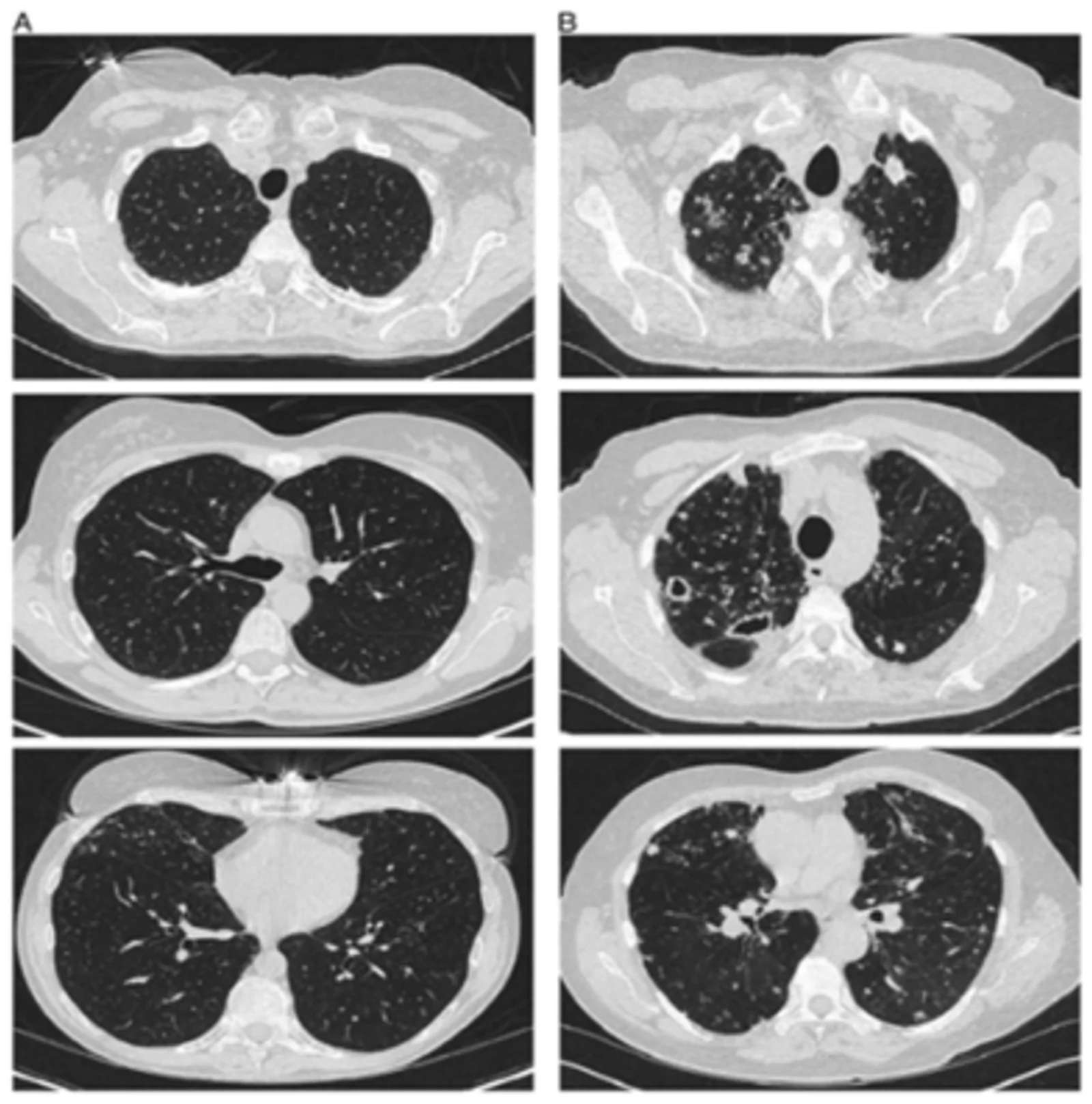
CT images showed: **(A)** Representative CT images of an NTM-C patient. Mild tubular bronchiectasis involving right middle and lower lobes and left lower lobe as well as nodular involvement of the corresponding lobes (Reiff score 3). **(B)** Representative CT images of an NTM-PD patient, characterized by 2 cavities in the right upper lobe and diffuse tubular bronchiectasis in all other lobes (Reiff score 8). There are nodules of varying sizes involving all lobes of lungs (37–39). CT, Computed Tomography; MDR-TB, Multi-drug resistant TB; NTM-C, Non-tuberculous mycobacteria colonization; NTM-PD, Non-tuberculous mycobacteria pulmonary diseases.

There are some also radiological tests like high resolution CT (HRCT), which plays a supportive role in evaluating suspected NTM pulmonary disease. HCRT alone cannot confirm NTM but studies showed that features like tree-in-bud nodules, consolidation, and atelectasis significantly improve diagnostic specificity for NTM (37). Early cavity or consolidation on CT may predict disease progression in nodular- bronchiectatic MAC cases (38). So, it can provide patterns ranging from FC changes in structurally damaged post-TB lungs to nodular–bronchiectatic involvement in milder disease forms. HRCT offers valuable diagnostic information; however, it inevitably has its limitations. Findings such as tree-in-bud nodules, consolidation, and atelectasis can be observed in both NTM and TB cases. The FC and nodular bronchiectatic (NB) types are the two main radiological patterns of non-tuberculous mycobacterial pulmonary disease (NTM-PD). A key differentiation is that the post-TB FC-type NTM disease is often found in older aged men with pre-existing pulmonary conditions (like COPD or previous TB disease), while the NB-type without TB history typically affects postmenopausal, non-smoking women with intrinsic susceptibility due to poor cell mediated immunity (37–39) (Figures 2, 3).

Novel diagnostic approaches, including serological testing for glycopeptidolipids and dual skin tests, are under investigation to improve detection sensitivity for NTM (19). Next-generation sequencing (NGS) has revolutionized diagnostics by enabling rapid and precise species identification, as well as detection of resistance genes directly from clinical specimens (40). NGS also provides insight into the airway microbiome, resistance evolution, and treatment response prediction-laying the foundation for personalized therapy in NTM pulmonary disease.

In South Asia, molecular assays are increasingly improving NTM detection beyond conventional culture. Indian LPA assays have shown clear utility in differentiating M. tuberculosis from major NTM species in bronchiectasis cohorts, reducing misclassification (41). Multi-gene sequencing approaches also demonstrate high diagnostic yield; in a recent evaluation of 59 isolates, sequencing positivity reached 89.8% for 16S rRNA and combined 16S+rpoB analysis achieved the highest agreement with MALDI-TOF MS (kappa 0.76) (42). Together with Indian data showing wider adoption of PCR-based identification, these findings highlight the growing epidemiological relevance of molecular detection in TB-endemic regions.

## 7 Diagnostic gaps in South Asia

Accurate detection of NTM-associated bronchiectasis remains a major challenge across South Asian countries due to limitations in laboratory infrastructure, inadequate diagnostic algorithms, and frequent misclassification as TB (Table 3). Most diagnostic laboratories in this region still depend on smear microscopy and conventional culture methods, which are insufficient for species-level identification or for differentiating NTM from *M. tuberculosis* (40, 43, 44). As a result, a significant proportion of NTM infections are either overlooked or incorrectly treated as drug-resistant TB, leading to delayed therapy, poor clinical outcomes, and unnecessary exposure to anti-tubercular drugs. This misdiagnosis is particularly problematic in patients with structural lung diseases like bronchiectasis, where chronic airway colonization by NTM often mimics active TB both clinically and radiologically (40, 45).

**Table 3:** Diagnostic lacuna of NTM in South Asia.

**Category**	**Current scenario in south Asia**	**Key Diagnostic Gaps**	**Impact on NTM–Bronchiectasis Management**
Diagnostic tools	Smear microscopy and culture widely used; molecular tools limited to tertiary labs	Lack of species level identification, Turnaround time is more	Causes misdiagnosis as TB which delayed treatment initiation (41)
Laboratory capacity	Few BSL-3 labs and limited sequencing facilities	Limited access outside urban centers	Under-detection, underreporting, poor surveillance (39)
Clinical awareness	NTM rarely suspected initially	Often considered only after failed TB therapy	Delayed diagnosis and inappropriate treatment (40)
Rapidly Growing Mycobacteria (RGM)	Frequently dismissed as contaminants	Absence of RGM-specific diagnostics	Missed infections and inadequate therapy (42)
Surveillance and reporting	Fragmented data, minimal environmental monitoring	No NTM integration in TB programs	Poor epidemiological understanding, no targeted policies (41)
Cost and accessibility	Most molecular tests and species-level diagnostics are expensive and limited to tertiary centers	Out-of-pocket costs, lack of government support, no inclusion in national TB programs	Patients skip testing, delayed diagnosis, and poor surveillance data (40)

The diagnostic landscape is further complicated by the limited availability of advanced molecular tools such as sequencing, line probe assays, and MALDI-TOF mass spectrometry, which remain largely restricted to specialized or tertiary care centers (43–45). The peripheral and district-level laboratories, where the majority of patient first present, often lacks the capacity for species-level identification or drug susceptibility testing, resulting in under-diagnosis and underreporting of NTM disease. Additionally, RGM, which are clinically significant in bronchiectasis progression, are frequently dismissed as contaminants due to insufficient diagnostic capabilities, despite their distinct therapeutic implications (40, 42, 43).

Another critical gap is the persistent lack of clinical awareness and structured surveillance systems. In many south Asian countries NTM-PD infections are only diagnosed after multiple time failure of anti-TB regimens and many physicians rarely consider NTM in cases of chronic or recurrent pulmonary disease (40, 43). Furthermore, National TB programs typically do not integrate NTM detection into their workflows, and environmental surveillance data remain scarce, despite strong evidence linking water and soil exposure to NTM transmission. This absence of epidemiological data limits understanding of species distribution, regional prevalence, and resistance patterns, thereby hindering the development of tailored diagnostic strategies and public health policies.

## 8 Treatment and emerging therapies

Treatment of NTM lung disease, particularly MAC, is prolonged, costly, and often associated with significant drug-related toxicities. Standard therapy requires a three-drug macrolide-based regimen including a rifamycin, ethambutol, and a macrolide, given either daily or intermittently depending on disease severity (46). Recent advances in understanding the microbiology and host–pathogen interaction of NTM have led to the development of new therapeutic modalities. The initiation of inhaled amikacin liposome inhalation suspension (ALIS) represents one of the most significant innovations for patients with refractory MAC disease. Randomized controlled trials demonstrated that ALIS, when added to guideline-based therapy, significantly increased sputum culture conversion rates in treatment-refractory MAC pulmonary disease (47). ALIS has now been approved by the U.S. FDA for this indication, offering a targeted delivery system that enhances drug concentration at the site of infection while minimizing systemic toxicity (48). The *M. kansasii* and *M. xenopi* infections tend to have more favorable outcomes when treated with rifampicin- and macrolide-based regimens (9, 47). In high TB-burden Asian settings, many patients with NTM-associated bronchiectasis initially receive empirical anti-TB therapy because CBNAAT and smear microscopy cannot differentiate MTBC from NTM. This leads to delayed initiation of appropriate regimens and worsening lung destruction. Severe drug intolerance and incomplete treatment adherence are common due to the prolonged regimen and limited counseling support in resource-limited centers.

Beyond antibiotics, bacteriophage therapy has emerged as a promising adjunctive approach for multidrug-resistant *M. abscessus* and *M. avium* infections. Despite guidelines based multidrug therapy, treatment outcomes in MAC associated lung diseases remain suboptimal, with high recurrence rates and significant toxicity concerns (49). Recent advances focus on optimizing macrolide-based regimens and incorporating inhaled or parenteral amikacin for refractory disease to improve culture conversion and limit resistance. Alternative and emerging antimicrobials, including clofazimine and bedaquiline, show promise in difficult-to-treat cases through synergistic bactericidal activity, although controlled clinical evidence is still limited (50). Early compassionate-use case reports have shown improvement in culture conversion and clinical outcomes with the use of specific mycobacteriophages, although standardized protocols and regulatory approvals remain pending (26). Immunomodulatory strategies are gaining interest, particularly given the role of host immune dysfunction in NTM persistence. Agents such as interferon-gamma, vitamin D supplementation, and targeted cytokine modulators are under investigation to enhance macrophage activation and improve pathogen clearance (12). Macrolides, aside from their antimicrobial effect, continue to serve as immunomodulators reducing neutrophilic inflammation in bronchiectatic lungs (47). Access to species-specific drugs such as ALIS, clofazimine, macrolides, and bedaquiline is highly restricted due to regulatory constraints, high cost, and lack of inclusion in existing national program ie. National Tuberculosis Elimination Program in India.

Treatment outcome may also vary according to the underlying disease etiology. Patients with post-TB structural lung disease or fibro cavitary involvement often require longer, more intensive regimens and show lower treatment success, whereas those with localized nodular bronchiectatic disease tend to respond more favorable to standard macrolide based therapy (31, 51). Recognizing these etiological differences helps clinicians individualize therapy beyond standard recommendations.

## 9 Bidirectional pathogenesis: a vicious cycle between NTM and bronchiectasis

The pathogenesis of NTM-PD in the context of bronchiectasis is a multifactorial process driven by complex interactions between host airway structure, immune defense, microbial adaptation, and environmental exposure (21). Among these, bronchiectasis characterized by irreversible airway dilation, mucus stasis, and impaired mucociliary clearance which plays a pivotal role in creating a permissive niche for NTM colonization and persistence (9, 52). In this way it creates favorable structural conditions for NTM colonization, but only a proportion of patients progress to active diseases (32). This heterogeneity is influenced by host susceptibility factors, environmental exposure, and pathogen specific virulence rather than bronchiectasis alone. Thus, bronchiectasis serves as a permissive condition but is not sufficient by itself for NTM-PD development.

The relationship between NTM and bronchiectasis is inherently bidirectional, forming a pathological cycle in which each condition predisposes to and amplifies the other. This interaction is increasingly recognized as a critical determinant of disease persistence, severity, and clinical outcomes. NTM are ubiquitous environmental organisms that opportunistically infect the airways, particularly in hosts with structural lung abnormalities. Whereas, bronchiectasis is characterized by irreversible airway dilation and impaired mucociliary clearance so it provides a niche environment where inhaled NTM can adhere, colonize, and evade host defense mechanism (53). The impaired clearance of mucus and reduced ciliary function facilitate bacterial persistence and biofilm formation, allowing NTM to resist both immune-mediated clearance and antimicrobial therapy (52). Mechanistically, bronchiectasis predisposes to NTM acquisition through impaired mucociliary clearance, chronic neutrophilic inflammation, and airway distortion that promotes biofilm formation (32, 54). NTM infection leads to epithelial injury and cytokine driven granulomatous inflammation, accelerating airway remodeling and worsening bronchiectasis (46).

**Figure 4:**
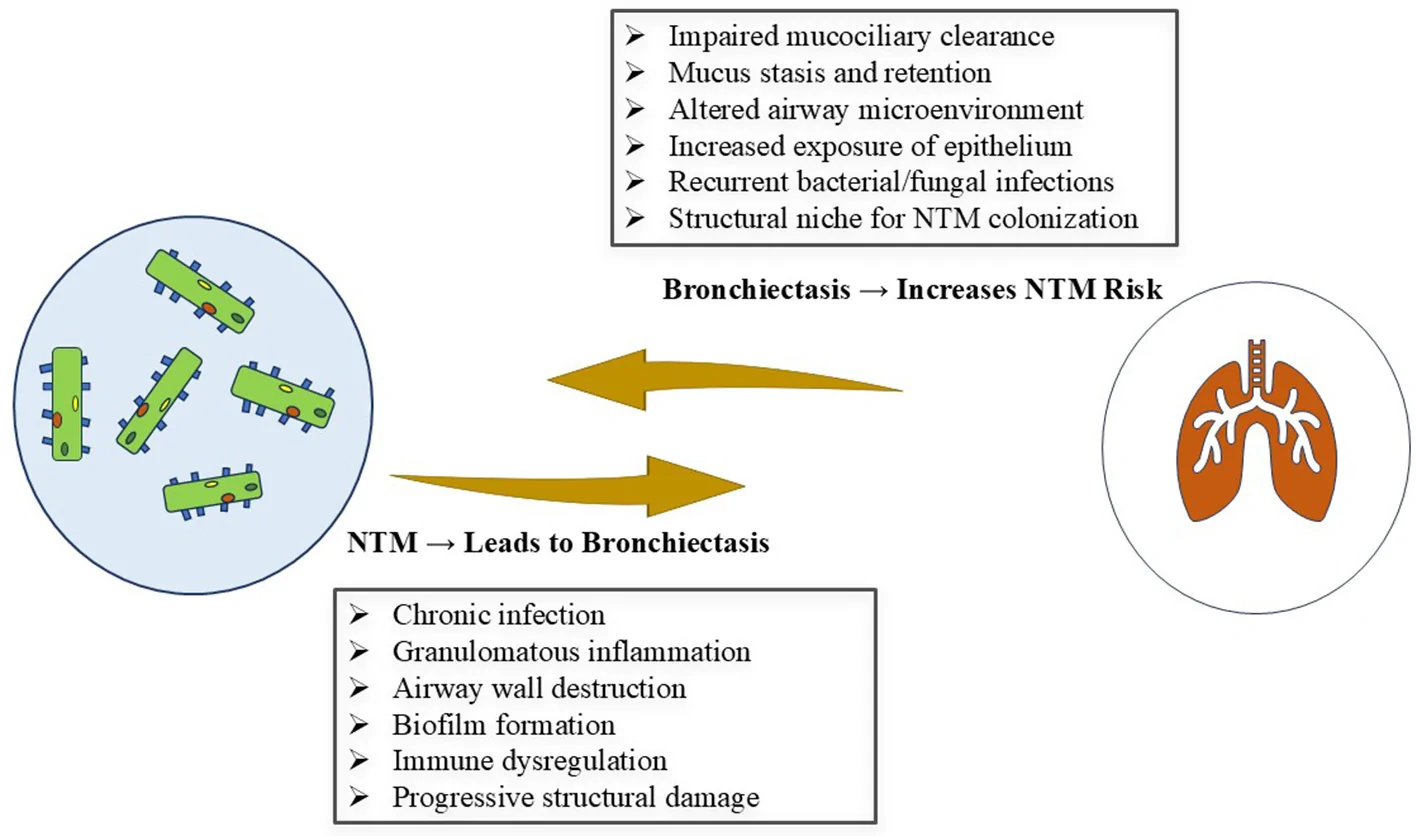
Diagram illustrates the bidirectional relationship between NTM infection and bronchiectasis of vicious cycle model. An individual predisposes for NTM infection due to Bronchiectasis by impaired mucociliary clearance, mucus stasis and retention, altered airway microenvironment, increased epithelial exposure, recurrent bacterial/fungal infections, and the creation of structural niches for NTM colonization. Conversely, NTM infection can contribute to the development of bronchiectasis by inducing chronic infection, granulomatous inflammation, airway wall destruction, biofilm formation, immune dysregulation, and progressive structural damage (9, 32, 51).

Chronic NTM infection, in turn, drives sustained inflammation and tissue destruction through cytokine release and granulomatous reactions, causing further architectural distortion of the airways and progression of bronchiectasis (55). Clinical evidence strongly supports this vicious cycle model (Figure 4). A significant proportion of bronchiectasis patients which is ranging from 9% to 50% that harbor active NTM infections, while conversely, NTM infection itself can initiate or accelerate bronchiectatic changes (9, 51). Repeated episodes of infection promote epithelial damage, mucus hypersecretion, and immune dysregulation, perpetuating airway remodeling (9, 32). This cycle is further compounded by the fact that immune suppression, use of inhaled corticosteroids, and co-existing conditions such as COPD or cystic fibrosis (CF) substantially increase susceptibility to both bronchiectasis and NTM-PD (55).

Biofilm formation by NTM within bronchiectatic airways not only enhances persistence but also facilitates horizontal gene transfer, potentially increasing antimicrobial resistance and complicating treatment outcomes (52). These resilient microbial communities stimulate chronic neutrophilic inflammation, leading to progressive lung destruction (51). Over time, the interplay between host structural vulnerability and pathogen adaptation shapes a feedback loop that is clinically evident as worsening lung function, recurrent exacerbations, and increased mortality (54). Understanding this bidirectional pathogenic loop is vital for clinical management, as interventions targeting only the infection or the structural disease in isolation are unlikely to achieve optimal outcomes. A comprehensive approach, combining aggressive antimicrobial therapy, airway clearance strategies, and modulation of inflammation, is essential to interrupt this cycle and improve patient prognosis.

## 10 Disease prognosis

The prognosis of NTM-PD is heterogenous in nature and it is influenced by numerous factors like host immune status, lung abnormalities, infecting species and co-morbid conditions (9, 12). This causes stability in mortality and relapse rate especially in patients with advanced bronchiectasis, cavitary lesions, or immunosuppression and also underscore the chronic and progressive nature of the disease (32). A nationwide Korean study have shown that bronchiectasis patients were 19-fold higher risk of developing NTM-PD compared with the general population which highlighted the persistent vulnerability and recurrent nature of infection (12). The prognosis varies markedly by species like as MAC infections demonstrate moderate responses to macrolide-based therapy, with sputum culture conversion rates ranging from 50% to 65%, while *M. abscessus* complex infections are notoriously difficult to treat, often requiring prolonged multidrug regimens including parenteral aminoglycosides and associated with relapse rates exceeding 40% (47, 52). Some studies have also shown that same species have sub-species level variation in antimicrobial susceptibility patterns which influence outcomes, for example, inducible macrolide resistance in *M. abscessus* subsp. *abscessus* contributing to higher relapse rates and lower cure rates than other sub-species (52).

Several clinical and radiological parameters serve as predictors of poor prognosis, including cavitary disease, bilateral involvement, extensive nodular–bronchiectatic patterns, low body mass index, and delayed sputum culture conversion (5, 9). Chronic infection and persistent airway inflammation contribute to progressive lung function decline over time, even in patients who achieve microbiological clearance (32). Relapse is frequently driven by reinfection with new NTM strains rather than reactivation of the original isolate, reflecting continuous environmental exposure and incomplete host defense (2, 56, 57). The long-term clinical burden extends beyond microbial persistence. Due to chronic cough, sputum production, fatigue and frequent exacerbations, patients experience sustained quality of life impairment (12, 57). In such a condition, there is requirement of multip-sectorial approaches, nutritional and psychological care to mitigate disease progression and improve patient outcomes.

## 11 Conclusion

Our review has shown that there is a cyclic loop between bronchiectasis and NTM-PD and for breaking this loop, targeted antimycobacterial therapy is required. For this purpose, regular surveillance of bronchiectasis patient is needed as they are more compromised by NTM. But many studies have shown that there are diagnostic gaps due to misdiagnosis of NTM as TB and limited laboratory infrastructure especially in south Asian regions. Some studies have also shown that reinfection with new NTM species is more common among pre-existing chronic lung diseases or bronchiectasis. So, multidisciplinary approaches and management are required to fill the diagnostic gaps. There is need to identify NTM infection early in immunocompromised patients or patients earlier infected with TB as they are more vulnerable to NTM-PD infection. Establishment of species-specific surveillance system integrating NTM testing into National TB Elimination Programme and enhancing clinician awareness will be key steps for reduction in misdiagnosed cases and improvement of patients' outcome. Advancement in molecular technology especially multiplex-PCR and sequencing will further enable in precision of medicine approaches ensuring timely intervention and better therapeutic success in managing NTM associated bronchiectasis.

## References

[1] Pathak K Hart S Lande L. Nontuberculous mycobacteria lung disease (NTM-LD): current recommendations on diagnosis, treatment, and patient management. Int J Gen Med. (2022) 15:7619–29. doi: 10.2147/IJGM.S272690 36213301 PMC9534142

[2] Ratnatunga CN Lutzky VP Kupz A Doolan DL Reid DW Field M . The rise of non-tuberculosis mycobacterial lung disease. Front Immunol. (2020) 11:303. doi: 10.3389/fimmu.2020.00303 32194556 PMC7062685

[3] Zhou Y Mu W Zhang J Wen SW Pakhale S. Global prevalence of non-tuberculous mycobacteria in adults with non-cystic fibrosis bronchiectasis 2006-2021: a systematic review and meta-analysis. BMJ Open. (2022) 12:e055672. doi: 10.1136/bmjopen-2021-055672 35914904 PMC9345037

[4] Prados Sánchez C González G Quirós Fernández S Martínez Redondo M Mangas Moro A. Nontuberculous mycobacteria and bronchiectasis. Community Acquir Infect. (2016) 3:104. doi: 10.4103/2225-6482.198489

[5] Shteinberg M. Waterer G Chotirmall SH. A Global effort to stop the vicious vortex: a special american journal of respiratory and critical care medicine issue for world bronchiectasis day 2024. Am J Respir Crit Care Med. (2024) 210:1–3. doi: 10.1164/rccm.202405-0947ED 38780090 PMC11197068

[6] Umrao J Singh D Zia A Saxena S Sarsaiya S Singh S . Prevalence and species spectrum of both pulmonary and extrapulmonary nontuberculous mycobacteria isolates at a tertiary care center. Int J Mycobacteriol. (2016) 5:288–93. doi: 10.1016/j.ijmyco.2016.06.008 27847012

[7] Singh AK Maurya AK Umrao J Kant S Kushwaha RAS Nag VL . Role of GenoType^®^ mycobacterium common mycobacteria/additional species assay for rapid differentiation between *Mycobacterium tuberculosis* complex and different species of non-tuberculous mycobacteria. J Lab Physicians. (2013) 5:083–9. doi: 10.4103/0974-2727.119847 24701099 PMC3968636

[8] Van Braeckel E Bosteels C. Growing from common ground: nontuberculous mycobacteria and bronchiectasis. Eur Respir Rev. (2024) 33:2024. doi: 10.1183/16000617.0058-2024 38960614 PMC11220627

[9] Fujita M. Pulmonary nontuberculous mycobacteria infection in bronchiectasis: a narrative review of current status and future. Health Sci Rep. (2025) 8:e70749. doi: 10.1002/hsr2.70749 40276131 PMC12018276

[10] Prieto MD Alam ME Franciosi AN Quon BS. Global burden of nontuberculous mycobacteria in the cystic fibrosis population: a systematic review and meta-analysis. ERJ Open Res. (2023) 9:00336-2022. doi: 10.1183/23120541.00336-2022 36605902 PMC9808535

[11] Prevots DR Marshall JE Wagner D Morimoto K. Global epidemiology of nontuberculous mycobacterial pulmonary disease: a review. Clin Chest Med. (2023) 44:675–721. doi: 10.1016/j.ccm.2023.08.012 37890910 PMC10625169

[12] Choi H Xu JF Chotirmall SH Chalmers JD Morgan LC Dhar R. Bronchiectasis in Asia: a review of current status and challenges. Eur Respir Rev. (2024) 33:1–12. doi: 10.1183/16000617.0096-2024 39322263 PMC11423131

[13] Yadav S Rawal G. Understanding the spectrum and management of post-tuberculosis lung disease: a comprehensive review. Cureus. (2024) 16:e63420. doi: 10.7759/cureus.63420 39077302 PMC11284254

[14] Jing C Zheng H Wang X Wang Y Zhao Y Liu S . Disease burden of tuberculosis and post - tuberculosis in Inner Mongolia, China, 2016 – 2018. — based on the disease burden of post - TB caused by COPD. BMC Infect Dis. (2023) 23:406. doi: 10.1186/s12879-023-08375-w 37316793 PMC10265833

[15] Chin KL Sarmiento ME Alvarez-Cabrera N Norazmi MN Acosta A. Pulmonary non-tuberculous mycobacterial infections: current state and future management. Eur J Clin Microbiol Infect Dis. (2020) 39:799–826. doi: 10.1007/s10096-019-03771-0 31853742 PMC7222044

[16] Kumar K Ponnuswamy A Capstick TG Chen C McCabe D Hurst R . Non-tuberculous mycobacterial pulmonary disease (NTM-PD): epidemiology, diagnosis and multidisciplinary management. Clin Med J R Coll Physicians London. (2024) 24:100017. doi: 10.1016/j.clinme.2024.100017 38387207 PMC11024839

[17] Frajman A Izhakian S Mekiten O Hadar O Lichtenstadt A Hajaj C . Phenotypical characteristics of nontuberculous mycobacterial infection in patients with bronchiectasis. Respir Res. (2024) 25:1–8. doi: 10.1186/s12931-024-02904-0 39010067 PMC11251292

[18] Yoon SH Kim HJ Kim J Kim J Lee JH. Nontuberculous mycobacterial pulmonary disease presenting as bronchiolitis pattern on CT without cavity or bronchiectasis. BMC Pulm Med. (2024) 24:432. doi: 10.1186/s12890-024-03223-2 39223547 PMC11367750

[19] Hendrix C McCrary M Hou R Abate G. Diagnosis and management of pulmonary NTM with a focus on *Mycobacterium avium* complex and *Mycobacterium abscessus*: challenges and prospects. Microorganisms. (2023) 11:47. doi: 10.3390/microorganisms11010047 36677340 PMC9861392

[20] Retuerto-Guerrero M López-Medrano R de Freitas-González E Rivero-Lezcano OM. Nontuberculous mycobacteria, mucociliary clearance, and bronchiectasis. Microorganisms. (2024) 12:1–15. doi: 10.3390/microorganisms12040665 38674609 PMC11052484

[21] Lira RL de S Nogueira FAB Campos R de FP de C Ferreira DRM Roxo PLBT de Azevedo CCS . *Mycobacterium abscessus* subsp. massiliense: biofilm formation, host immune response, and therapeutic strategies. Microorganisms. (2025) 13:447. doi: 10.3390/microorganisms13020447 40005812 PMC11858063

[22] Weiss CH Glassroth J. Pulmonary disease caused by nontuberculous mycobacteria. Expert Rev Respir Med. (2012) 6:597–613. doi: 10.1586/ers.12.58 23234447

[23] Wang PH Shu CC Sheu CC Chang CL Hsieh MH Hsu WH . Clinical predictors of nontuberculous mycobacteria lung disease and coisolates of potential pathogenic microorganisms in noncystic fibrosis bronchiectasis. Open Forum Infect Dis. (2024) 11:1–8. doi: 10.1093/ofid/ofae427 39145140 PMC11322833

[24] Ojo O Odeyemi A. Non-mycobacteria tuberculosis in Africa: a literature review. Ethiop J Health Sci. (2023) 33:913–8. doi: 10.4314/ejhs.v33i5.21 38784502 PMC11111205

[25] Bjerrum S Oliver-Commey J Kenu E Lartey M Newman MJ Addo KK . Tuberculosis and non-tuberculous mycobacteria among HIV-infected individuals in Ghana. Trop Med Int Heal. (2016) 21:1181–90. doi: 10.1111/tmi.12749 27383726

[26] Martinez-Garcia MA. The heterogeneous world of nontuberculous mycobacteria in bronchiectasis. Am J Respir Crit Care Med. (2024) 210:18–20. doi: 10.1164/rccm.202405-0936ED 38747644 PMC11197064

[27] Simons S van Ingen J Hsueh PR Van Hung N Dekhuijzen PN Boeree MJ . Nontuberculous mycobacteria in respiratory tract infections, eastern Asia. Emerg Infect Dis. (2011) 17:343–9. doi: 10.3201/eid1703.100604 21392422 PMC3165997

[28] Suresh P Kumar A Biswas R Vijayakumar D Thulasidharan S Anjaneyan G . Epidemiology of nontuberculous mycobacterial infection in tuberculosis suspects. Am J Trop Med Hyg. (2021) 105:1335–8. doi: 10.4269/ajtmh.21-0095 34424857 PMC8592207

[29] Moon P Guillaumin E. Chan ED. Non-tuberculous mycobacterial lung disease due to multiple “minor” risk factors: an illustrative case and a review of these “lesser elements”. J Thorac Dis. (2020) 12:4960–72. doi: 10.21037/jtd-20-986 33145070 PMC7578471

[30] Rout SS Turuk J Dm NS Giri S K A Kumar S . Non-tuberculosis mycobacterial infection among clinically suspected tuberculosis in eastern India (2019–2023). J Infect Public Health. (2025) 18:102888. doi: 10.1016/j.jiph.2025.102888 40627902

[31] Griffith DE Aksamit TR. Bronchiectasis and Nontuberculous Mycobacterial Disease. Clin Chest Med. (2016) 33:283–95. doi: 10.1016/j.ccm.2012.02.002 22640846

[32] Cowman S van Ingen J Loebinger MR Griffith DE. Non-tuberculous mycobacterial pulmonary disease. Eur Respir J. (2019) 54:1900250. doi: 10.1183/13993003.00250-2019 31221809

[33] Huang HL Lee MR Liu CJ Cheng MH Lu PL Wang JY . Predictors of radiographic progression for NTM–pulmonary disease diagnosed by bronchoscopy. Respir Med. (2020) 161:105847. doi: 10.1016/j.rmed.2019.105847 31785506

[34] Gardini G Ori M Codecasa LR Matteelli A. Pulmonary nontuberculous mycobacterial infections and environmental factors: a review of the literature. Respir Med. (2021) 189:106660. doi: 10.1016/j.rmed.2021.106660 34715617

[35] Ryu YJ Koh WJ Daley CL. Diagnosis and treatment of nontuberculous mycobacterial lung disease: clinicians' perspectives. Tuberc Respir Dis (Seoul). (2016) 79:74–84. doi: 10.4046/trd.2016.79.2.74 27066084 PMC4823187

[36] Zhang H Tang M Li D Xu M Ao Y Lin L. Applications and advances in molecular diagnostics: revolutionizing non-tuberculous mycobacteria species and subspecies identification. Front Public Heal. (2024) 12:1410672. doi: 10.3389/fpubh.2024.1410672 38962772 PMC11220129

[37] Kwak N Lee CH Lee H ju Kang YA Lee JH Han SK . Non-tuberculous mycobacterial lung disease : diagnosis based on computed tomography of the chest. Eur Radiol. (2016) 26:4449–56. doi: 10.1007/s00330-016-4286-6 26945763

[38] Lee G Lee KS Moon JW Koh WJ Jeong BH Jeong YJ . Nodular bronchiectatic Mycobacterium avium complex pulmonary disease. Natural course on serial computed tomographic scans. Ann Am Thorac Soc. (2013) 10:299–306. doi: 10.1513/AnnalsATS.201303-062OC 23952847

[39] Garcia B Wilmskoetter J Grady A Mingora C Dorman S Flume P. Chest computed tomography features of nontuberculous mycobacterial pulmonary disease versus asymptomatic colonization: a cross-sectional cohort study. J Thorac Imaging. (2022) 37:140–5. doi: 10.1097/RTI.0000000000000610 34292274

[40] Rajendran P Padmapriyadarsini C Mondal R. Nontuberculous mycobacterium: an emerging pathogen: Indian perspective. Int J Mycobacteriol. (2021) 10:217–27. doi: 10.4103/ijmy.ijmy_141_21 34494559

[41] Murthy MK Gupta VK Maurya AP. Diagnosis of nontuberculous mycobacterial infections using genomics and artificial intelligence-machine learning approaches: scope, progress and challenges. Front Microbiol. (2025) 16:1665685. doi: 10.3389/fmicb.2025.1665685 40969435 PMC12440979

[42] Rodriguez-pazmiño AS Carvajal E Echeverr J Paredes-nu D Calderon J Orlando SA . Comparative evaluation of MALDI-ToF mass spectrometry and Sanger sequencing of the 16S, hsp65, and rpoB genes for non tuberculous mycobacteria species identi fi cation. Front Cell Infect Microbiol. (2025) 15:1612459. doi: 10.3389/fcimb.2025.1612459 40792107 PMC12336121

[43] Chindam A Vengaldas S Reddy V Syed U Kilaru H Prasad N . Journal of clinical tuberculosis and other mycobacterial diseases challenges of diagnosing and treating non-tuberculous mycobacterial pulmonary disease [NTM-PD]: a case series. J Clin Tuberc Other Mycobact Dis. (2021) 25:100271. doi: 10.1016/j.jctube.2021.100271 34541338 PMC8441069

[44] Kangabam N Hegadi S Divya C Venkatarayappa N Murthy K. Prevalence, challenges in diagnosis and treatment of non-tuberculous mycobacteria. Appl Microbiol Theor Technol. (2025) 6:6274. doi: 10.37256/amtt.6120256402

[45] Shrivastava K Kumar C Singh A Narang A Giri A Sharma NK . An overview of pulmonary infections due to rapidly growing mycobacteria in South Asia and impressions from a subtropical region. Int J Mycobacteriol. (2020) 9:6270. doi: 10.4103/ijmy.ijmy_179_19 32474491

[46] Aksamit TR Philley J V Griffith DE. Nontuberculous mycobacterial (NTM) lung disease: the top ten essentials. Respir Med. (2014) 108:417–25. doi: 10.1016/j.rmed.2013.09.014 24484653

[47] Daley CL. Winthrop KL. *Mycobacterium avium* complex : addressing gaps in diagnosis and management. J Infect Dis. (2020) 222:199–211. doi: 10.1093/infdis/jiaa354 PMC7566660 32814943

[48] Loukeri AA Papathanassiou E Kavvada A Kampolis CF Pantazopoulos I Moschos C . Amikacin liposomal inhalation suspension for non-tuberculous mycobacteria lung infection: a greek observational study. Medicina. (2024) 60:1–11. doi: 10.3390/medicina60101620 39459407 PMC11509699

[49] Chung C. Current and emerging treatment strategies for *Mycobacterium avium* complex pulmonary disease: a narrative review. Ewha Med J. (2025) 48:e25. doi: 10.12771/emj.2025.00080 40703365 PMC12277505

[50] Johnson TM Byrd TF Drummond WK Childs-Kean LM Mahoney M V Pearson JC . Contemporary pharmacotherapies for nontuberculosis mycobacterial infections: a narrative review. Infect Dis Ther. (2023) 12:343–65. doi: 10.1007/s40121-022-00750-5 36609820 PMC9925655

[51] Larsson L. olof Polverino E Hoefsloot W Codecasa LR Diel R Jenkins SG . Expert review of respiratory medicine pulmonary disease by non-tuberculous mycobacteria – clinical management, unmet needs and future perspectives. Expert Rev Respir Med. (2017) 11:977–89. doi: 10.1080/17476348.2017.1386563 28967797

[52] Philley J V DeGroote MA Honda JR Chan MM Kasperbauer S Walter ND . Erratum to: treatment of non-tuberculous mycobacterial lung disease. Curr Treat Options Infect Dis. (2016) 8:297–8. doi: 10.1007/s40506-016-0102-8 PMC5436303 28529461

[53] Long MB Chotirmall SH Shteinberg M Chalmers JD. Rethinking bronchiectasis as an inflammatory disease. Lancet Respir Med. (2024) 12:901–14. doi: 10.1016/S2213-2600(24)00176-0 38971168

[54] Chalmers JD Aksamit T Carvalho ACC Rendon A Franco I. Non-tuberculous mycobacterial pulmonary infections. Pulmonology. (2018) 24:120–31. doi: 10.1016/j.pulmoe.2017.12.005

[55] Dhasmana DJ Whitaker P van der Laan R Frost F. A practical guide to the diagnosis and management of suspected Non-tuberculous Mycobacterial Pulmonary Disease (NTM-PD) in the United Kingdom. NPJ Prim Care Respir Med. (2024) 34:45. doi: 10.1038/s41533-024-00403-9 39709516 PMC11663218

[56] Jhun BW Kim SY Moon SM Jeon K Kwon OJ Huh HJ . Development of macrolide resistance and reinfection in refractory *Mycobacterium avium* complex lung disease. Am J Respir Crit Care Med. (2018) 198:1322–30. doi: 10.1164/rccm.201802-0321OC 29877739

[57] Stout JE Koh WJ Yew WW. Update on pulmonary disease due to non-tuberculous mycobacteria. Int J Infect Dis. (2016) 45:123–34. doi: 10.1016/j.ijid.2016.03.006 26976549

